# Social support and psychological safety in university volleyball: a qualitative study of sustained participation

**DOI:** 10.3389/fpsyg.2026.1837354

**Published:** 2026-05-14

**Authors:** Huiteng Xu, Tiantian Hu

**Affiliations:** Physical Education Department, Woosuk University, Jeonju, Republic of Korea

**Keywords:** Education for Sustainable Development (ESD), learning ecosystem, physical education, qualitative research, social support, sustainable learning

## Abstract

**Purpose:**

Grounded in the framework of Education for Sustainable Development (ESD), this study explores how social support contributes to the construction of a sustainable learning ecosystem in university physical education. Focusing on a volleyball elective course, it examines the mechanisms through which teacher support, peer support, and course/organizational support enhance students' psychological safety, interest, self-efficacy, and sustained participation, thereby promoting skill development.

**Method:**

A qualitative research design was adopted. Semi-structured interviews were conducted with volleyball instructors, undergraduate students, and course/organizational stakeholders (*N* = 35). The interview data were analyzed via the coding procedures of constructivist grounded theory.

**Findings:**

(1) Teacher support demonstrates a “dual-mode” pattern, integrating structured professional guidance with emotional care, jointly facilitating skill acquisition and psychological safety; (2) Peer support exhibits a “resonance mode,” enhancing collaboration, classroom belonging, and sustained participation through cognitive stimulation and emotional resonance; (3) Course and organizational support establish a psychologically safe environment and provide conditions for long-term development, enabling social support to be internalized into students' sustained participation intentions.

**Conclusion:**

Sustained participation in university volleyball courses is achieved through an interactive learning ecosystem jointly shaped by teacher, peer, and organizational support. External support is closely associated with stable participation experiences, as reflected in participants' accounts of enhanced psychological safety, increased learning confidence, and sustained engagement. This study highlights the integrative role of multidimensional social support in sustainable learning and provides theoretical implications for ESD-oriented curriculum design in university physical education. The study does not claim causal mechanisms but instead interprets participants' perceived experiences of support within the volleyball learning context.

## 1 Introduction

In the context of higher education, physical education (PE) courses are not only sites for skill training, but also important educational settings that promote students' psychological wellbeing, social development, and sustained engagement in learning (Han et al., 2025). Compared with academic courses, university PE integrates physical practice, emotional experience, and social interaction, making learning outcomes highly sensitive to interpersonal relationships and contextual support (Lin et al., 2025). Among these, volleyball elective courses, characterized by technical complexity, frequent peer comparison, and public performance, create a challenging learning environment. Such an environment may stimulate motivation, but it may also induce learning anxiety due to the public nature of errors and evaluative pressure, particularly among beginners (Bao, 2025). These factors may have a negative impact on learning motivation and sustained participation. Thus, effective learning in university volleyball courses cannot be explained solely by instructional techniques or individual ability, but is closely related to social interaction and the supportive environment within the learning context. Social support is regarded as an important contextual resource influencing learning motivation, self-efficacy, and emotional regulation (Martínez-López et al., 2023). Current studies have shown that teacher and peer support are closely associated with learning engagement and psychological wellbeing in general educational settings. However, most of this research has focused on academic contexts and has examined relationships between variables using quantitative methods. In contrast, within physical education context—where bodily performance, immediate interaction, and peer collaboration are central—there remains a lack of process-oriented explanations of how social support is experienced by students, how it is enacted in interaction, and how different types of support jointly shape students' learning experiences and participation processes (Schüller et al., 2025). In particular, in university PE courses, teacher, peer, and course/organizational support are intertwined, jointly constituting a supportive environment that influences students' learning experiences (Ruzek et al., 2016). Although previous studies have confirmed the positive associations between teacher support, peer support, and students' engagement or wellbeing in general educational and physical education contexts [e.g., SDT-based motivation and engagement studies (Ruzek et al., 2016)], these studies have predominantly examined linear relationships using quantitative designs in general educational or non-sport contexts. In university physical education—especially volleyball courses characterized by public performance and immediate peer visibility—how these forms of support are actually experienced, negotiated, and jointly constructed in interaction remains underexplored. Moreover, existing research has rarely distinguished the context-specific mechanisms in East Asian higher education PE settings, where collectivist learning cultures and performance sensitivity may shape support perception differently (Han et al., 2025). Therefore, the novelty of this study is not limited to combining established constructs, but lies in revealing how multidimensional social support is experienced as an interactional process within volleyball learning environments, and how these lived experiences relate to psychological safety and sustained participation.

Psychological safety refers to learners' subjective perception that they can express ideas, try new approaches, and learn from mistakes without fear of ridicule or negative evaluation (Korneeva et al., 2022). In physical education contexts, psychological safety is particularly important, as skill learning is often accompanied by performance failure, public exposure, and immediate peer evaluation. When students perceive the environment as supportive, they are more likely to actively attempt new techniques, express learning difficulties, and engage in teamwork. However, existing research on psychological safety has primarily focused on organizational behavior and general classroom contexts, and lacks systematic explanations of how psychological safety is constructed through teacher support and peer interaction in physical skill learning environments (Han et al., 2022). Sustainable Development Goal 4.7 emphasizes inclusive and learner-centered education that promotes lifelong learning and individual wellbeing (UNESCO, 2020). Under sufficient support conditions, university volleyball courses can not only enhance motor skills, but also foster long-term participation in physical activity and positive attitudes toward sport. Therefore, it is necessary to examine, within specific instructional contexts, how social support is experienced in classroom interaction and how it relates to students' psychological safety and sustained participation.

Based on this, this study adopts a qualitative research design to explore the forms of teacher support, peer support, and course/organizational support in classroom interaction within university volleyball elective courses, and how these forms of support are associated with students' experiences of psychological safety and sustained participation. The study addresses the following research questions: from a theoretical perspective, Education for Sustainable Development (ESD) is used as an interpretive lens rather than a strict operational framework, helping to understand how inclusive interaction, participation continuity, and supportive learning environments are enacted in physical education contexts.

RQ1: How do participants perceive and experience teacher support, peer support, and course/organizational support in university volleyball elective courses?

RQ2: In the process of volleyball learning, how do teacher support and peer support interact, and how are they related to students' emotional experiences and psychological safety?

RQ3: In what ways are multiple forms of social support associated with students' sustained participation?

## 2 Literature review

### 2.1 Social support in university physical education

Social support is an important contextual factor influencing learners' motivation, sustained participation, and psychological wellbeing (Luo et al., 2025). In general educational research, teacher and peer support have been shown to be closely associated with positive learning emotions, self-efficacy, and sustained engagement (Guo et al., 2025). Supportive social environments shape students' subjective perceptions of learning contexts through emotional regulation, enhanced sense of belonging, and tolerance of learning-related risks, thereby influencing learning behavior and sustained participation (Collie, 2024). In physical education contexts, the importance of social support is particularly salient due to the embodied, public, and emotional nature of learning (Kruse et al., 2024). Students in university PE courses are required to confront performance success and failure, peer comparison, and physical challenges, making them more sensitive to interpersonal feedback and environmental characteristics (Su and Liu, 2025). Compared with academic classrooms, physical skill learning involves immediate performance and public evaluation, which may increase students' sense of uncertainty and evaluative anxiety (Zhong et al., 2024). Therefore, learning experiences are influenced not only by instructional content, but also by how teachers respond to errors, how peers interact, and the overall level of environmental support (Charteris et al., 2021).

When students consistently perceive positive responses, supportive interactions help reduce perceived psychological risk and lay the foundation for a safe and inclusive learning environment (McGuire, 2025). In this sense, social support is not merely an external resource, but a contextual condition experienced and constructed by learners through interaction (Peng et al., 2025), which profoundly influences their willingness to attempt new skills, express difficulties, and sustain participation (Held and Mori, 2024). Therefore, it is important to further examine, within specific classroom contexts, how different types of social support are perceived in interaction and how they jointly influence learning experiences.

### 2.2 Multidimensional attributes of teacher and peer support

Teacher support is a multidimensional construct encompassing emotional support, instructional support, and autonomy support (Sun et al., 2025). Emotional support emphasizes empathy, encouragement, and sensitivity to students' emotional states (Zhang and Hu, 2025); instructional support focuses on facilitating skill acquisition through demonstration, feedback, and scaffolding (Chang and Yang, 2023); and autonomy support highlights learners' agency, choice, and self-regulation in the learning process (Li and Zeng, 2025). In university volleyball courses, these forms of support typically coexist and are intertwined in practice (Franco et al., 2025). Students require technical guidance when learning complex skills, emotional support when facing repeated failure, and autonomy to adjust their learning pace according to their own conditions. Existing research indicates that autonomy support contributes to enhanced motivation and sustained participation in physical education contexts (Mammadov, 2023). However, how these forms of support are enacted in classroom interaction and interpreted by students still requires further exploration from an experiential perspective.

In addition, peer support represents another key dimension of the physical learning environment (Zhang and Han, 2026). Physical education is grounded in cooperative practice and interactive engagement, where peers play a central role. Emotional peer support can alleviate learning anxiety and reduce concerns about making mistakes (Flick et al., 2024), while learning-oriented peer support promotes skill acquisition through observation, reminders, and informal feedback (Metz et al., 2024). Continuous peer interaction also contributes to the development of classroom belonging and enhances learning engagement (Van Kessel et al., 2025). In volleyball courses, students complete learning tasks through cooperative practice, encouragement, and experience sharing. Peer interaction not only influences skill acquisition, but also shapes students' overall perceptions of the classroom climate. Importantly, teacher support and peer support do not operate independently; rather, they function together within specific instructional contexts, forming an ongoing interaction process (Lee et al., 2022). This interaction may reduce perceived risk in learning situations, thereby encouraging students to attempt new movements, express difficulties, and maintain participation. Therefore, understanding the synergistic effects of different forms of support from an interactional perspective is of particular importance.

### 2.3 Psychological safety in physical learning contexts

Psychological safety is an important concept for explaining how social contexts influence learning behavior (Bump and Cladis, 2025). Originally developed in organizational research, it refers to individuals' subjective perception that engaging in interpersonal risk-taking behaviors (such as making mistakes or expressing uncertainty) will not lead to negative consequences (Moffett et al., 2024). In educational settings, psychological safety is closely associated with classroom participation, exploratory behavior, and willingness to engage in challenging tasks (McLeod and Gupta, 2023). When learners perceive the environment as supportive, they are more likely to express difficulties, actively try new strategies, and participate in interaction (Hu et al., 2026). In physical education contexts, psychological safety is particularly important due to the performative, public, and evaluative nature of skill learning (Taylor et al., 2021). Students are required to demonstrate movements in front of teachers and peers, and skill acquisition is often accompanied by repeated failure and adjustment. When the classroom environment is perceived as unsafe, students may protect themselves by avoiding participation or reducing engagement (Senel et al., 2024). In contrast, when the environment tolerates mistakes and emphasizes the learning process, students are more likely to maintain exploratory behavior and demonstrate greater persistence (Elbe et al., 2021).

Existing research highlights that teacher and peer support are key sources influencing psychological safety in physical education classrooms (Adinata et al., 2025). Teachers' responses to errors, peer interaction styles, and the overall classroom climate jointly shape students' perceptions of the learning environment (Frazier et al., 2017). When students consistently experience understanding, encouragement, and collaborative interaction, they are more likely to perceive the environment as psychologically safe, thereby sustaining participation (Robinson and Held, 2025). However, there remains a lack of process-oriented explanations of how such support is experienced in specific classroom interactions and how it relates to psychological safety.

### 2.4 Multilevel support and sustained participation

In recent years, educational research has shifted from single-variable approaches toward more contextualized perspectives, emphasizing that learning experiences are shaped through the ongoing interaction among individuals, social relationships, and institutional environments (Jornet et al., 2025; Konstantinidis, 2024). This perspective is particularly relevant in physical education contexts, where learning processes are highly dependent on teacher guidance, peer interaction, and course organization. In classroom practice, teacher support, peer support, and course/organizational support do not function independently, but jointly shape students' experiences of the learning environment through continuous interaction (Prananto et al., 2025; Saxer et al., 2024). For example, teachers' feedback approaches, peer interaction climates, and course norms (e.g., whether mistakes are tolerated or process-oriented learning is emphasized) may collectively influence students' perceptions of classroom risk, thereby affecting their participation behavior (Huang and Jeong, 2025; Zhou et al., 2023). On this basis, psychological safety can be understood as an important experiential condition linking external support and learning behavior. When students perceive lower interpersonal risk within a context of multiple forms of support, they are more likely to express difficulties, attempt new skills, and sustain engagement in the learning process. Therefore, psychological safety should not be viewed as an isolated construct, but as an experiential outcome embedded within specific support interactions.

However, existing research has largely adopted quantitative approaches to examine relationships between support and learning outcomes, with relatively limited attention to how different sources of support are experienced by students in specific instructional contexts, and how they relate to psychological safety and sustained participation. Therefore, it is necessary to adopt a qualitative approach to explore the operational processes of multiple forms of social support in physical education classrooms, and their relationships with students' learning experiences from a process-oriented perspective.

## 3 Methods

### 3.1 Research design

This study adopted a qualitative research design to explore how teacher support and peer support jointly operate in classroom interaction within university volleyball courses, and how they are associated with students' psychological safety and sustained participation. As the study focuses on the interactional processes and meaning construction of social support, qualitative methods are particularly suitable for capturing experiences and interactional dynamics in complex learning contexts (Bates et al., 2025). Methodologically, this study is positioned as a constructivist grounded theory-informed qualitative inquiry rather than a fully formal grounded theory study, emphasizing iterative interpretation of participants' meaning-making processes within context.

In addition, the study drew on the analytical principles of constructivist grounded theory during the data analysis process (Lindqvist and Forsberg, 2023). Through constant comparison and conceptual abstraction, the interactive experiences of multiple forms of social support in physical education classrooms were systematically analyzed to generate process-oriented explanations. This approach emphasizes the co-construction of meaning between researchers and participants, and focuses on the relationships among action, interaction, and contextual understanding. From a theoretical perspective, a holistic view of learning contexts was adopted (Biesta, 2020), conceptualizing physical education learning as a process jointly shaped by individual experience, social interaction, and the classroom environment. The detailed interview protocol and coding procedures are provided in the Supplementary Tables S1–S3.

### 3.2 Research participants

This study employed purposive sampling (Campbell et al., 2020) to recruit teachers and students from volleyball elective courses offered at three comprehensive universities in South Korea. Sampling and analysis were conducted iteratively, with early interview insights informing subsequent participant selection and refinement of interview focus. A total of 35 participants were included (Table 1): (1) 16 volleyball instructors representing different career stages and teaching roles; (2) 16 undergraduate students with varying academic years, volleyball experience, and levels of course participation; and (3) three course- and organization-related staffs, providing institutional and organizational perspectives.

**Table 1:** Sampling framework and participant characteristics.

Group	Dimension	Categories	*n*	Purpose
Volleyball teachers (*N* = 16)	Teaching experience	≤ 5 years (5); 6–15 years (6); >15 years (5)	16	Capture developmental differences in teaching beliefs and support practices
	Teaching role	Main course instructors (8); team coaches (4); PE school teachers (4)	16	Ensure diversity of instructional contexts
Students (*N* = 16)	Academic year	Year 1–4 (balanced distribution)	16	Capture learning stage differences
	Skill level	Experienced (7); beginners (9)	16	Reflect variation in competence
	Engagement level	High (10); low/irregular (6)	16	Ensure heterogeneity of participation
Stakeholders (*N* = 3)	Role	Administrator (1); coordinator (1); support staff (1)	3	Triangulate institutional perspective

These three participant groups correspond to the primary sources of teacher support, peer support, and organizational support. By integrating data from multiple perspectives, the study aims to comprehensively understand how different types of support are manifested in classroom interaction and how they are experienced by participants. To ensure confidentiality and analytical clarity, all participants were anonymized using coded identifiers: teachers (T1–T16), students (S1–S16), and organizational staff (OS1–OS3). Detailed sampling criteria and group distributions are presented in Supplementary Table S1. The aim of sampling was not statistical representativeness but to achieve theoretical saturation and capture diverse experiential perspectives across participant roles.

### 3.3 Data collection

This study employed semi-structured interviews for data collection (DeJonckheere and Vaughn, 2019). This method allows for thematic focus while providing participants with sufficient space to express their experiences and construct meaning, making it particularly suitable for exploring the mechanisms linking social support, psychological safety, and sustained participation in physical education contexts. The interview protocol was developed based on relevant literature and maintained openness and flexibility during implementation to allow for the emergence of new experiential themes (Table 2). The interviews covered five dimensions: (1) Teacher support: emotional care, constructive feedback, and autonomy support; (2) Peer support: collaborative learning, encouragement, and learning assistance; (3) Course and organizational support: teaching facilities, course arrangements, and classroom norms; (4) Psychological safety: concerns about making mistakes, willingness to express difficulties, and attempts to try new skills; and (5) Sustained participation: cognitive perceptions, emotional experiences, and long-term participation intentions regarding the volleyball course.

**Table 2:** Semi-structured interview guide.

Dimension	Student questions	Teacher/staff questions
Warm-up	Describe a memorable volleyball learning experience	Common student challenges in volleyball courses
Teacher support	How does the teacher respond to mistakes?	How do you balance instruction and emotion?
Peer support	How do peers affect your confidence?	What peer behaviors support learning most?
Organizational support	How do you evaluate course environment?	How does curriculum support learning?
Psychological safety	Do you fear making mistakes in class?	How do you create a safe climate?
Learning outcomes	How has volleyball affected you?	How does support influence long-term participation?

Each interview lasted approximately 40 min. All participants provided informed consent prior to participation. The interviews were audio-recorded and transcribed verbatim. Interviews were conducted in Korean, and all transcripts were translated into English by the research team. A back-checking procedure was applied to ensure semantic consistency between original and translated versions. The interviews were conducted by the first author, who has prior experience in qualitative sport pedagogy research and was familiar with the university PE context, while maintaining a non-teaching and non-evaluative role during data collection. NVivo 12 was used to assist with data coding and analysis. The full interview protocol is presented in Supplementary Table S2.

### 3.4 Data analysis

This study drew on the analytical procedures of constructivist grounded theory (Charmaz, 2006) and conducted data analysis through three stages: open coding, axial coding, and selective coding (three-level coding) (Strauss and Corbin, 1998). To enhance analytical transparency, in addition to anonymized participant identifiers, a coding system was introduced to distinguish conceptual categories at different levels. Lowercase letters with numbers (e.g., a1, c1) represent initial concepts generated during open coding, while uppercase letters with numbers (e.g., A1–A3, B1–B3, C1–C3) represent thematic categories developed during axial coding, corresponding respectively to different dimensions of teacher support, peer support, and course/organizational support (Table 3). Detailed coding procedures, examples, code definitions, and representative quotations are provided in Supplementary Tables S3–S7.

**Table 3:** Constructivist grounded theory coding process.

Stage	Category	Definition	Example	Function
Open coding	Initial concepts (a1–c1)	Meaning units from interviews	“Teacher didn't criticize me publicly…”	Concept generation
Axial coding	Categories (A1–C3)	Grouping support dimensions	Emotional/instructional/peer support	Category formation
Selective coding	Core category	Psychological safety & sustained participation	Integrated learning process model	Theory construction

The three coding stages were conducted as follows: (1) Open coding: Interview transcripts were analyzed line by line to identify initial concepts related to classroom interaction, support experiences, and learning experiences, resulting in 128 preliminary codes; (2) Axial coding: Through constant comparison, initial concepts were integrated and linked to form categories reflecting teacher support, peer interaction, and learning experiences; and (3) Selective coding: Core themes were further integrated through cross-category comparison to present the processual relationships between multiple forms of social support and learning experiences.

Beyond categorization, the analysis focused on identifying relationships among categories through constant comparison. The final process model emerged by linking these categories into a relational structure that explains how different forms of support are experienced and internalized. This emphasis on relational integration distinguishes the analysis from descriptive thematic coding and aligns with the theory-generating purpose of constructivist grounded theory.

To ensure research rigor, several strategies were adopted (Lincoln and Guba, 1985): (1) Member checking: Preliminary findings were shared with selected participants to confirm consistency with their experiences; (2) Inter-coder reliability: Two researchers independently coded approximately 30% of the transcripts and reached consensus through discussion; (3) Theoretical saturation: Data collection continued until no new concepts or significant thematic variations emerged; and (4) Reflexive memos: Analytical decisions and theoretical insights were documented throughout the research process to enhance transparency.

## 4 Findings

Based on the interview data, this study analyzed the manifestations of teacher support, peer support, and course/organizational support within classroom interaction experiences in university volleyball courses. Through constant comparison and inductive analysis, three major experiential themes were identified: (1) the multidimensional manifestations of teacher support; (2) the interactional characteristics of peer support; and (3) the role of course and organizational support in shaping the learning context. The coding structure of each theme and representative quotations are presented in Table 4 and Supplementary Tables S4–S7.

**Table 4:** Integrated themes of social support in university volleyball learning.

Support type	Dimension	Meaning	Representative quotation
Teacher support	Emotional	Reduces anxiety, builds safety	“The teacher comforted me before practice.” (S4)
	Instructional	Supports skill learning	“He demonstrated step by step.” (S1)
	Autonomy	Encourages self-paced learning	“I practiced at my own rhythm.” (S14)
Peer support	Emotional	Reduces fear of failure	“I wasn't afraid when practicing with classmates.” (S8)
	Cognitive	Provides learning assistance	“A classmate helped correct my movement.” (S11)
	Belonging	Builds classroom cohesion	“It felt like a friendly class.” (S16)
Organizational support	Resources	Ensures stable practice conditions	“Enough equipment made class smooth.” (OS3)
	Climate	Allows mistakes	“Making mistakes is normal.” (OS1)
	Development	Encourages lifelong sport	“Volleyball became lifelong exercise.” (OS2)

### 4.1 Multidimensional patterns of teacher support

#### 4.1.1 Emotional teacher support

Emotional support from teachers plays a critical role in university volleyball learning. Teachers not only provide emotional reassurance prior to technical instruction, but also enhance students' psychological safety by understanding anxiety, offering encouragement, and attending to students' emotional states (S4, A1; S7, a1).

S4 noted: “Before teaching the movement, the teacher encouraged me to stay relaxed, and only then started the demonstration” (a1). T3 similarly stated: “During competition preparation, I focus on helping students regulate their psychological state rather than only their technique” (A1). Such individualized attention enables students to maintain emotional stability and remain willing to try when facing technical challenges or mistakes. Emotional support is also reflected in ongoing companionship and trust building. S7 explained: “Both the teacher and classmates understand me. Even if I make mistakes, no one laughs at me, so I am more willing to keep trying.” T7 added: “Some students feel very nervous during training, so I adjust movements with them instead of criticizing them” (A1). These findings indicate that emotional teacher support not only alleviates learning anxiety but also contributes to the development of psychological safety, thereby supporting skill development and sustained participation. Through diverse forms of emotional support, teachers establish trustful relationships that continue to influence technical training, teamwork, and individual expression.

#### 4.1.2 Professional and instructional support

Teachers provide professional support through skill instruction and learning guidance. By breaking down movements, demonstrating step-by-step goals, and offering individualized feedback, teachers guide students to progressively master volleyball techniques (S1, A2; S5, A2).

S1 stated: “The teacher explained each movement step by step, and I quickly learned the serving technique” (A2). T1 noted: “I observe students' practice and provide targeted feedback at each stage to help them understand key points” (A2). This structured guidance improves skill acquisition and strengthens students' self-efficacy. Students also emphasized the importance of continuous feedback and appropriate challenge. S5 explained: “The teacher pointed out my mistakes but also showed me how to improve, which made me more confident each time” (A2). T6 highlighted: “Through progressive difficulty levels, students can complete tasks under manageable pressure and experience a sense of achievement” (A2). Professional support extends beyond technical instruction to include tactical training and game-based practice. T8 stated: “We simulate real match scenarios so students can apply their skills in practice.” S9 noted that this combination of practice and feedback supports both skill development and psychological adaptation (c1).

#### 4.1.3 Autonomy-supportive teaching

In autonomy-supportive teaching, teachers encourage students to make choices, regulate their learning pace, and explore personal interests. S14 stated: “I can practice serving and receiving at my own pace, which motivates me to try new techniques” (A3). As T4 noted: “In creative tasks, I provide a framework but allow students to design their own practice and performance forms” (A3). Such practices provide greater participation space and support sustained engagement. Autonomy support also facilitates personalized skill development and creative exploration. S12 stated: “Choosing my own practice helped me discover my preferred passing style and try different tactics” (A3). Similarly, T9 emphasized: “Students explore independently while I ensure safety and provide feedback” (A3). This approach enhances engagement and contributes to psychological safety, supporting long-term participation and skill internalization.

### 4.2 Interactional characteristics of peer support

#### 4.2.1 Emotional peer support

Emotional peer support provides psychological buffering during volleyball learning. Students reported receiving companionship, encouragement, and reassurance during mistakes or competitive stress (S8, B1; S12, B1). S8 stated: “When practicing with others, I feel supported and less afraid of making mistakes” (B1). S12 also noted: “When I feel nervous, my peers make me feel less alone” (B1). This emotional resonance reduces anxiety and strengthens persistence and classroom belonging. Over time, such interactions form stable social support that underpins psychological safety.

#### 4.2.2 Learning assistance

Peer learning support occurs through reminders, demonstrations, and shared experiences (S11, B2; S6, B2). S11 stated: “My classmates remind me of movement details so I can correct them in time” (B2). S6 added: “When I face difficulties, we think of solutions together, so I don't feel alone” (B2). These interactions promote skill acquisition and collaborative learning strategies. They also support both cognitive and emotional development, enabling students to attempt new skills with greater confidence.

#### 4.2.3 Sense of classroom belonging

Sustained peer interaction enhances students' sense of belonging (S16, B3). S16 noted: “The classroom atmosphere gradually became very friendly, and everyone helped each other” (B3). S1 added: “In group activities, we progress together, which motivates me” (B3). However, not all participants reported uniformly positive experiences. One student noted a contrasting experience: “I was afraid of being laughed at, so I didn't dare to practice in front of others” (S3), indicating that peer presence may also generate performance anxiety in certain contexts. This pattern is further supported by multiple participant accounts. For example, S8 described that “with someone practicing with me, I wasn't that afraid,” highlighting the emotional buffering function of peer interaction. Similarly, S16 noted that “gradually, it felt like a very friendly class,” indicating how continuous peer interaction contributes to classroom belonging and sustained engagement. A strong sense of belonging reduces fear of failure and evaluation, contributing to psychological safety and supporting continued participation. Notably, differences in perception were observed across participant groups. While teachers tended to emphasize instructional structure and skill progression, students—particularly beginners—more frequently highlighted emotional reassurance and peer companionship as central to their participation experience. These contrasts further underscore the relational and context-dependent nature of social support in physical education.

### 4.3 Course and organizational support

#### 4.3.1 Course and resource support

Adequate facilities, structured course arrangements, and teaching resources provide essential support for learning (OS3, C1). OS3 noted: “Sufficient equipment allows the course to run smoothly” (C1). Such structural support creates a stable learning environment, enabling students to focus on practice and reducing resource-related anxiety.

#### 4.3.2 Psychological safety climate

At the organizational level, psychological safety is fostered through clear norms and encouragement of trial and error (OS1, C2). OS1 stated: “Teachers emphasize that making mistakes is normal and part of learning” (C2). This climate, combined with teacher and peer support, encourages students to attempt new skills and engage in challenging tasks.

#### 4.3.3 Learning and development support

Organizational support promotes long-term participation and wellbeing (OS2, C3). OS2 stated: “I am now more willing to see volleyball as a lifelong activity” (C3). Such support reinforces students' interest and self-efficacy, contributing to sustained participation.

### 4.4 Processual relationships between support and sustained participation

The findings indicate that teacher, peer, and organizational support operate differently across learning stages. Teacher support is particularly salient in reducing uncertainty at early stages, while peer support becomes increasingly important for maintaining participation. Organizational support provides a stable contextual foundation. These support experiences are closely related to students' perceptions of the classroom environment: when support involves understanding, encouragement, and shared responsibility, students are more likely to perceive the classroom as a space where they can safely attempt participation; repeated success experiences and feedback enhance confidence and interest; and sustained support contributes to stable participation habits. Importantly, this process is not linear but involves ongoing interaction and reinforcement.

The process model presented in this study was not predefined but inductively developed through iterative comparison across coding stages. Specifically, recurring linkages between categories—such as teacher emotional support, peer interaction, and students' expressions of reduced fear and increased willingness to participate—were consistently identified across participants. These patterned associations provided the empirical basis for integrating individual categories into a coherent process framework, rather than a purely descriptive thematic summary. Overall, multiple forms of social support collectively construct a learning context that influences students' behavioral willingness, particularly their readiness to attempt new skills, express difficulties, and sustain participation (A1–A3; B1–B3; C1–C3). These relationships were supported by multiple participant quotations across groups, reflecting consistent experiential patterns rather than isolated observations. Across participants, variability was observed in how support was perceived; beginners tended to emphasize emotional reassurance, whereas more experienced students highlighted instructional feedback and autonomy as more influential.

**Figure 1:**
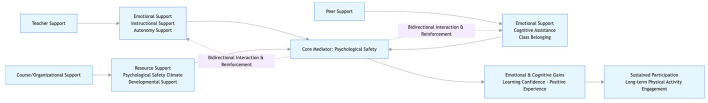
A process model of support systems, psychological safety, and sustained participation in university volleyball learning.

Figure 1 presents the processual relationships among teacher support, peer support, and course/organizational support in university volleyball learning. It shows how multiple forms of support are associated with students' psychological safety, emotional experience and confidence, and sustained participation through an ongoing process of interaction and reinforcement. The figure was reconstructed by the authors based on grounded coding categories and reflects an interpretive synthesis rather than a predefined theoretical model.

## 5 Discussion

### 5.1 Multidimensional features of teacher support

The findings indicate that teacher support in university volleyball courses is primarily manifested in three interrelated dimensions: emotional support, professional (instructional) support, and autonomy support, which address RQ1 and RQ2. This finding is consistent with existing studies on the multidimensional structure of teacher support (Su and Liu, 2025; Mammadov, 2023; Prananto et al., 2025).

Professional and instructional support constitutes the foundation of students' skill development. Through movement decomposition, task scaffolding, situational simulation, and immediate feedback, teachers transform complex skills into more understandable and practicable learning tasks. This structured support not only improves the efficiency of skill acquisition, but also enhances students' confidence in their own abilities through gradual progress (Guo et al., 2025; Kruse et al., 2024; Huang and Jeong, 2025). Unlike traditional outcome-oriented teaching, this study shows that process-oriented guidance helps students develop a more stable sense of competence and reduces frustration caused by failure (Han et al., 2025; Kruse et al., 2024). Emotional support plays a key role in students' perception of psychological safety in the classroom. When students attempt new skills or face mistakes, teachers provide encouragement, empathy, and non-judgmental responses, which makes a potentially stressful learning environment more likely to be perceived as an acceptable space for exploration (Ruzek et al., 2016; Charteris et al., 2021; Frazier et al., 2017). Particularly in volleyball, which involves public performance and immediate evaluation, emotional support is perceived by students as a key protective factor against learning anxiety, particularly in relation to peer evaluation and the exposure of failure in publicly performed volleyball activities (Taylor et al., 2021; Senel et al., 2024). This finding further reveals that teacher support is not limited to skill transmission, but is closely related to how students interpret risk and error in the classroom.

Autonomy-supportive teaching provides conditions for more active student participation. By offering choices, allowing individual pacing, and designing open-ended tasks, teachers shift students from passive execution to more active engagement (Kruse et al., 2024; Held and Mori, 2024; Mammadov, 2023). In this process, students can adjust their learning paths according to their abilities and interests, thereby maintaining more stable participation. Autonomy support not only increases classroom engagement, but is also associated with students' long-term intention to participate (Su and Liu, 2025; Huang and Jeong, 2025). Overall, teacher support in university volleyball courses is not a single form, but a combination of structured guidance, emotional responsiveness, and autonomy provision. This finding is consistent with research on teacher support, self-determination theory, and motivation in physical education (Su and Liu, 2025; Mammadov, 2023; Huang and Jeong, 2025), and further indicates that in highly interactive and publicly evaluative physical learning contexts, emotional support plays a particularly important role in shaping students' perception of classroom risk (Hu et al., 2026; Adinata et al., 2025; Frazier et al., 2017).

### 5.2 Interactive features of peer support

Consistent with teacher support, peer support in volleyball courses is mainly reflected in three interconnected aspects: learning assistance, emotional support, and classroom belonging, further addressing RQ2. Learning assistance: Peers provide supplementary cognitive resources through observational demonstration, immediate reminders, and experience sharing. This informal feedback is immediate and incorporates the context of the moment, allowing students to continuously adjust their actions and strategies during practice (Bao, 2025; Zhou et al., 2023).

In addition, peer interaction not only supports skill acquisition, but also expands students' ways of understanding during practice. Emotional support: Encouragement and companionship from peers constitute a continuous source of emotional support. In repeated practice and competition contexts, students are more likely to receive immediate responses from peers. This shared experience of “facing challenges together” helps alleviate anxiety and frustration (Flick et al., 2024; Adinata et al., 2025). Compared with teacher support, peer support is often more routine and continuous, especially in each moment of practice, error, and adjustment. Sense of belonging: When students establish relatively stable relationships through collaborative practice and team activities, their perception of the classroom shifts from an individual performance context to a more supportive collective space. This sense of belonging reduces sensitivity to evaluation and enhances willingness to engage in complex tasks and try new skills (Van Kessel et al., 2025; Saxer et al., 2024). Therefore, peer support is not limited to emotional encouragement, but also includes cognitive assistance and relational belonging. This finding is consistent with social support theory and research on peer interaction in physical education (Martínez-López et al., 2023; Chen et al., 2026), and further suggests that peer support is not a peripheral factor, but an important classroom resource for sustaining participation (Zhou et al., 2023; Van Kessel et al., 2025).

### 5.3 Course and organizational support as contextual conditions

Course and organizational support constitute important contextual conditions for students' learning experiences and also respond to RQ1. The findings indicate that perceived support comes not only from interpersonal interactions with teachers and peers, but also from more stable environmental factors such as course design, resource allocation, and institutional norms (Martínez-López et al., 2023; Jornet et al., 2025).

Course and resource support provide the basic conditions for skill learning. Adequate facilities, reasonable grouping, and appropriate time arrangements enable students to practice repeatedly in a relatively stable environment, reducing uncertainty and anxiety caused by resource limitations. These conditions provide a practical foundation for effective teacher and peer interaction (Metz et al., 2024; Bates et al., 2025). Norms and classroom climate at the course level play an important role in the formation of psychological safety. When the classroom explicitly emphasizes that “making mistakes is part of learning” and restricts behaviors such as ridicule or negative evaluation, students are more likely to perceive the classroom as a space for trying and practicing. Therefore, psychological safety is not only an individual perception, but is also closely related to course norms and organizational climate (Charteris et al., 2021; Bump and Cladis, 2025; Frazier et al., 2017). Learning and developmental support is reflected in the emphasis on long-term participation in physical activity and psychological wellbeing. When courses focus not only on short-term skill training but also on health development and sustained participation, students are more likely to perceive volleyball learning as a long-term valuable activity rather than a temporary task (UNESCO, 2020; Konstantinidis, 2024). Thus, course and organizational support are not merely background factors, but important contextual conditions that work together with teacher and peer support. This finding supports the necessity of understanding physical education learning experiences from a holistic learning environment perspective and indicates that students' participation experiences are gradually enriched under multi-level support conditions (Peng et al., 2025; Jornet et al., 2025).

### 5.4 Support, psychological safety, and sustained participation

Integrating the three types of support to address RQ3, the findings indicate that teacher support, peer support, and course/organizational support do not operate independently, but jointly influence students' understanding of the learning environment in classroom interaction. Among these, psychological safety serves as an important experiential condition linking external support and sustained participation (Hu et al., 2026; Frazier et al., 2017).

When teachers provide encouragement, peers are willing to share pressure, and the course climate allows attempts and mistakes, students are more likely to perceive the classroom as a space where they “can try.” This perception of safety helps students express difficulties, attempt new movements, and reduce concerns about failure exposure (Charteris et al., 2021; Senel et al., 2024). Within such a relatively safe classroom experience, students are more likely to build confidence through repeated practice, positive feedback, and incremental success, thereby maintaining learning interest (Guo et al., 2025; Kruse et al., 2024). When students continuously experience support, controllability, and positive feedback, they are more likely to sustain participation and perceive volleyball courses as meaningful long-term learning activities (Su and Liu, 2025; Huang and Jeong, 2025). It should be noted that the purpose of this study is not to test causal relationships among variables, but to explain how multiple forms of social support are experienced in specific physical education contexts and how they relate to psychological safety and sustained participation. In this sense, the study responds to SDG 4.7's emphasis on inclusive and learner-centered education (UNESCO, 2020) and provides a more contextualized explanation of sustained participation in university physical education. It should be emphasized that the process model presented in this study is an interpretive synthesis grounded in participants' accounts rather than a verified causal mechanism.

From an ESD perspective, the three forms of social support identified in this study can be interpreted as concrete pedagogical enactments of sustainable learning principles. Teacher support reflects guided participation and developmental scaffolding, peer support embodies collaborative and inclusive interaction, and organizational support ensures stable and equitable learning conditions. In this sense, ESD functions not merely as a conceptual framing, but as an interpretive lens that explains how supportive environments foster sustainable engagement in physical education.

### 5.5 Theoretical contributions

This study contributes to existing research in several ways:

(1) It shows that in university volleyball courses, teacher support includes not only technical instruction but also emotional responsiveness and autonomy support, extending existing understandings of teacher support in physical education contexts characterized by public performance and immediate evaluation. Specifically, the study explains how psychological safety emerges through the interaction of teacher scaffolding, peer resonance, and organizational norms, rather than being treated as a pre-existing condition.
(2) It demonstrates that peer support involves not only emotional encouragement, but also learning assistance and the development of classroom belonging. Peer interaction functions both as an emotional resource and as a relational condition for sustaining participation.
(3) By integrating teacher support, peer support, and course/organizational support into a unified analytical framework, the study provides an interpretive framework for understanding how multi-level support is experienced in interaction and how it may relate to psychological safety and sustained participation in this specific context. Compared with studies focusing on single variables, this study emphasizes the interactive and contextual nature of support.
(4) From a process perspective, the study explains how external support is associated with psychological safety, confidence development, and sustained participation. This does not constitute a causal model, but provides a process-based explanation grounded in participants' experiences, offering implications for future longitudinal, mixed-methods, and intervention studies.

### 5.6 Limitations and future research

Although this study highlights the importance of teacher support, peer support, and course support in university volleyball learning, several limitations should be noted:

The research context is limited to volleyball, a competitive and team-based sport with high public performance characteristics, and participants' responses may be influenced by social desirability bias inherent in such visible and evaluative settings. Additionally, the collectivistic campus culture and PE curriculum system of Korean universities are context-specific, which may limit the transferability of the findings to other cultural settings or to individual-oriented sports contexts.

(1) The study adopts a cross-sectional qualitative design, which does not capture the dynamic changes of support, psychological safety, and sustained participation over time; (2) the sample is limited to Korean universities, and the transferability of the findings to other cultural and educational contexts requires further examination. In addition, the study context is shaped by a single-sport (volleyball) environment in Korean universities, where collectivist classroom culture and performance-oriented PE practices may influence how social support and psychological safety are experienced. These contextual features further limit the direct transferability of findings to other cultural or sport settings; (3) the data are mainly based on interviews, lacking triangulation with classroom observation, behavioral data, or multi-method evidence. Because the data rely primarily on self-reported interviews, the findings may also be influenced by social desirability bias and retrospective interpretation; and (4) the study focuses on micro-level classroom interaction, with relatively limited attention to broader institutional and policy-level factors.

Future research should: (1) adopt longitudinal, diary-based, or mixed-methods designs to capture dynamic processes; (2) extend to different national and institutional contexts to test transferability; (3) incorporate classroom observation, learning records, or psychological measures to enhance ecological validity; and (4) apply multi-level analysis to examine interactions between macro-level structures and micro-level psychosocial processes. The reference list was carefully reviewed, and duplicate citations and weakly aligned interdisciplinary references were removed or replaced with more directly relevant physical education and sport pedagogy literature to improve disciplinary coherence.

## 6 Conclusion

From the perspective of Education for Sustainable Development, this study employs a qualitative design to examine how teacher support, peer support, and course/organizational support are related to students' psychological safety and sustained participation in university volleyball courses. The findings indicate that sustained participation in volleyball learning is not the result of a single factor, but is gradually formed through multi-level support conditions: (1) Teacher support is mainly reflected in professional guidance, emotional responsiveness, and autonomy support; (2) Peer support is reflected in learning assistance, emotional support, and classroom belonging; and (3) Course and organizational support provide stable contextual conditions through resource provision, normative climate, and developmental orientation. These three types of support do not operate independently, but jointly shape students' understanding of the learning environment. When students perceive the classroom as a space where they can try, make mistakes, and receive support, they are more likely to maintain confidence, sustain interest, and continue participating in volleyball learning. This study responds to the emphasis of SDG 4.7 on inclusive and learner-centered education, and provides practical implications for transforming university physical education from skill-oriented training to more supportive, inclusive, and sustainable learning environments.

## Data Availability

The original contributions presented in the study are included in the article/Supplementary material, further inquiries can be directed to the corresponding author.
