# Supplementary material for: Social support and psychological safety in university volleyball: a qualitative study of sustained participation
Source: Front Psychol. 2026 May 14;17:1837354. doi: 10.3389/fpsyg.2026.1837354 (PMC13216176; doi:10.3389/fpsyg.2026.1837354)
Supplement: Supplementary file 1 [file Supplementary_file_1.docx]

**Table S1. Sampling framework and basic characteristics of qualitative research participants**

| **Group** | **Sampling dimension** | **Specific criteria** | **n** | **Purpose and rationale** |
| --- | --- | --- | --- | --- |
| Volleyball teachers (N = 16) | Years of teaching experience | Novice teachers  (≤ 5 years) | 5 | To cover different career stages and explore variations in support beliefs and teaching practices |
|  |  | Experienced teachers  (6–15 years) | 6 |  |
|  |  | Senior teachers  (> 15 years) | 5 |  |
|  | Teaching/coaching role | Main instructors of elective volleyball courses | 8 | To represent typical instructional contexts including classroom teaching, skill training, and assessment |
|  |  | University team or extracurricular training coaches | 4 | To supplement high-intensity, competition-oriented teaching contexts |
|  |  | Volleyball-related teachers in physical education schools | 4 | To reflect integrated instructional and developmental support practices |
|  | Teaching support style (pre-assessment) | Technical-oriented / Emotional-oriented / Autonomy-supportive | — | To ensure diversity in teacher support patterns (A1 emotional, A2 professional, A3 autonomy support) |
| University students (N = 16) | Academic year | Freshman to senior  (≈ 4 per year) | 16 | To examine changes in perceived support across different stages of university learning |
|  | Course participation | Regular attendance and training | 10 | To cover varied levels of engagement |
|  |  | Irregular attendance or low engagement | 6 |  |
|  | Volleyball background | With secondary-school volleyball experience | 7 | To explore differentiated support needs based on skill level |
|  |  | No prior experience / beginners | 9 |  |
|  | Support experience (pre-screening) | Positive / neutral / negative perceptions of teacher, peer, and course support | — | To capture diverse learning experiences and avoid sample homogeneity |
| Course & organizational stakeholders  (N = 3) | Functional role | Physical education department administrator | 1 | To triangulate organizational support from a management perspective |
|  |  | Volleyball course coordinator / teaching-research leader | 1 |  |
|  |  | Student sport and health support staff (e.g., counselor, school physician) | 1 | To supplement student support and safeguarding perspectives |

**Table S2. Core semi-structured interview guide**

| **Target dimension** | **Sample questions for students** | **Sample questions for teachers / stakeholders** |
| --- | --- | --- |
| Contextual warm-up & rapport building | Can you describe a volleyball class or training experience that left a strong impression on you? | What learning or psychological challenges do students commonly face in elective volleyball courses? |
| Teacher support experience | How does the teacher usually respond when you make mistakes during practice or assessment? | How do you balance technical requirements with emotional care and confidence building in teaching? |
|  | Are you allowed to adjust training intensity based on your physical or emotional condition? | How do you support students’ autonomy in practice and self-expression? |
| Peer support experience | How do classmates interact with you when you feel nervous, frustrated, or unable to keep up? | What peer support behaviors do you observe as most beneficial to learning? |
| Course & environmental support | How do you evaluate the course design, assessment methods, and learning atmosphere? | How do curriculum design and institutional policies reflect a student-centered approach? |
| Psychological safety | Do you ever hesitate to try new skills because of fear of making mistakes or being judged? | How do you foster a classroom atmosphere that allows mistakes and experimentation? |
| Learning meaning & development | How have these support experiences influenced your views on volleyball, exercise, or self-development? | How do supportive teaching practices influence students’ long-term sport participation and well-being? |
| Closing | Are there any other support experiences important to your volleyball learning that we did not discuss? | Are there additional factors crucial to building a supportive volleyball course environment? |

**Table S3. Three-level coding process of constructivist grounded theory**

| Analysis stage | Code name and definition | Representative quotations (participant ID) | Theoretical memo |
| --- | --- | --- | --- |
| Open coding | a1 Constructive responses after mistakes | “When I hit the ball out, the teacher didn’t criticize me publicly but talked to me afterward about how to adjust.” (S7) | Protects self-esteem and reduces classroom anxiety |
|  | a2 Negotiated adjustment of training intensity | “I told the teacher my legs were tired, and he let me reduce jump serves.” (T4) | Respects physical perception and autonomy |
|  | b1 Peer companionship during frustration | “I kept missing receptions, and my classmates stayed to practice with me.” (S12) | Emotional buffering function of peer support |
|  | c1 Lack of psychological safety (negative case) | “I was afraid of being laughed at, so I didn’t dare to practice.” (S9) | Highlights the importance of a safe climate |
| Axial coding | A1 Emotional teacher support | Teachers provide care through understanding, respect, and encouragement | Integration of multiple open codes |
|  | A3 Autonomy-supportive teaching | Teachers allow regulation and choice in learning |  |
|  | B1 Peer emotional support | Empathy and companionship among students |  |
|  | C2 Psychological safety climate | Classroom tolerance for mistakes and experimentation | Emerging core mechanism |
| Selective coding | Core category | Psychological safety and sustained participation in university volleyball learning empowered by a social support system | Integrates all categories |

**Table S4. Theme 1: Multidimensional pattern of teacher support**

| **Second-level code** | **Third-level code**  **(specific experiences)** | **Representative quotation** |
| --- | --- | --- |
| A1 Emotional teacher support | Private encouragement, understanding learning anxiety, attention to emotional changes | “The teacher comforted me first before teaching the movement.” (S4) |
| A2 Professional and learning support | Movement decomposition, targeted feedback, learning pathway guidance | “He demonstrated the movement slowly so I could follow.” (S1) |
| A3 Autonomy-supportive teaching | Soliciting practice feedback, allowing personalized learning pace | “I could practice at my own rhythm.” (S14) |

**Table S5. Theme 2: Synergistic patterns of peer support**

| **Second-level code** | **Third-level code**  **(specific experiences)** | **Representative quotation** |
| --- | --- | --- |
| B1 Emotional peer support | Encouragement, accompaniment, anxiety relief | “With someone practicing with me, I wasn’t that afraid.” (S8) |
| B2 Learning assistance | Skill reminders, peer demonstration, experience sharing | “One reminder from a classmate helped me correct it.” (S11) |
| B3 Classroom belonging | Group collaboration, shared progress, positive atmosphere | “Gradually, it felt like a very friendly class.” (S16) |

**Table S6. Theme 3: Empowering role of course and organizational support**

| **Second-level code** | **Third-level code**  **(specific experiences)** | **Representative quotation** |
| --- | --- | --- |
| C1 Course and resource support | Adequate facilities and equipment, reasonable scheduling | “There was enough equipment, so class felt smooth.” (OS3) |
| C2 Psychological safety climate | Permission to make mistakes, anti-mockery norms, positive feedback | The teacher said making mistakes is normal. (OS1) |
| C3 Learning and developmental support | Encouragement of long-term sport participation and well-being | Now I’m more willing to treat volleyball as a lifelong exercise. (OS2) |

**Table S7. Core category and generative mechanism**

| **Core category** | **Supportive category** | **Relational structure**  **(grounded theory articulation)** | **Generative outcomes** |
| --- | --- | --- | --- |
| Psychological safety and sustained participation in university volleyball learning empowered by a social support system | Teacher support  (A1 + A2 + A3) | Builds initial trust and reduces uncertainty | Enhanced confidence and participation intention |
|  | Peer support  (B1 + B2 + B3) | Maintains emotional engagement and classroom belonging | Increased engagement and persistence |
|  | Course and organizational support  (C1 + C2 + C3) | Provides structural conditions that legitimize supportive interactions | Formation of long-term sport participation |
